# 1520. Exploring the factors linked to prior HIV testing and schistosomiasis treatment in Malawi.

**DOI:** 10.1093/ofid/ofad500.1355

**Published:** 2023-11-27

**Authors:** Geoffrey K Kangogo

**Affiliations:** Saint Louis University, Saint Louis, Missouri

## Abstract

**Background:**

Fishing exposes fishermen to schistosomiasis-infested fresh water and concurrently through precarious livelihoods to risky sexual behaviour, rendering these two infections occupational hazards for fishermen. This study aimed to characterize the knowledge of the two conditions to obtain necessary data for a subsequent cluster randomized trial designed to investigate demand creation strategies for joint HIV-schistosomiasis service provision in fishing villages on the shores of southern Lake Malawi.

**Methods:**

Enumeration of all resident fishermen was carried out from 2019 to 2020. The fishermen reported their knowledge, attitudes and practices in the uptake of HIV and schistosomiasis services. Knowledge of HIV status, previous receipt of praziquantel and willingness to attend a beach clinic were modelled using random effects binomial regression, accounting for clustering.

**Results:**

A total of 6,297 fishermen were surveyed from the 45 clusters with harmonic mean number of fishermen per cluster of 112 (95% CI: 97; 134). The mean age was 31.7y (SD: 11.9) and nearly 40% (2,474/6,297) could not read or write. Overall, 1,334/6,293 (21.2%) had never tested for HIV, with 64.4% (3,191/4,956) having tested in the last 12 months, and 5.9% (373/6290) taking antiretroviral therapy (ART). Only 40% (1,733/4,465) had received praziquantel in the last 12 months. Every additional year of age was associated with 1% decreased likelihood of having taken praziquantel (aRR: 0.99, 95% CI: 0.98-0.99, p< 0.001). However, recent HIV testing increased the likelihood of taking praziquantel (aRR 2.24, 95% CI: 1.93-2.62, p< 0.001).

**Conclusion:**

In a setting with an underlying high prevalence of both HIV and schistosomiasis, we found low knowledge of HIV status and low utilization of free schistosomiasis treatment. Among fishermen who accessed HIV services, there was a very high likelihood of taking praziquantel suggesting that integrated service delivery may lead to good coverage.

**Disclosures:**

**All Authors**: No reported disclosures

